# Adult vultures outperform juveniles in challenging thermal soaring conditions

**DOI:** 10.1038/srep27865

**Published:** 2016-06-13

**Authors:** Roi Harel, Nir Horvitz, Ran Nathan

**Affiliations:** 1 Department of Ecology, Evolution and Behavior, Alexander Silberman Institute of Life Sciences, The Hebrew University of Jerusalem Edmond J. Safra Campus, Jerusalem 91904, Israel

## Abstract

Due to the potentially detrimental consequences of low performance in basic functional tasks, individuals are expected to improve performance with age and show the most marked changes during early stages of life. Soaring-gliding birds use rising-air columns (thermals) to reduce energy expenditure allocated to flight. We offer a framework to evaluate thermal soaring performance, and use GPS-tracking to study movements of Eurasian griffon vultures (*Gyps fulvus*). Because the location and intensity of thermals are variable, we hypothesized that soaring performance would improve with experience and predicted that the performance of inexperienced individuals (<2 months) would be inferior to that of experienced ones (>5 years). No differences were found in body characteristics, climb rates under low wind shear, and thermal selection, presumably due to vultures’ tendency to forage in mixed-age groups. Adults, however, outperformed juveniles in their ability to adjust fine-scale movements under challenging conditions, as juveniles had lower climb rates under intermediate wind shear, particularly on the lee-side of thermal columns. Juveniles were also less efficient along the route both in terms of time and energy. The consequences of these handicaps are probably exacerbated if juveniles lag behind adults in finding and approaching food.

Low performance in basic functional tasks can have detrimental consequences for individuals and might explain the relatively high mortality rates in juveniles often seen in nature^1^,^2^. Recently, several studies have focused on the effect of age on movement performance of birds over large scales, mostly during migration^3^,^4^,^5^. Yet, our knowledge on how exactly these differences are mediated and how experience affects movement performance on the most relevant small scale is limited^6^. Recent advances in technology have enabled tracking of free-ranging animals at high spatial and temporal resolutions while gathering detailed information about their behavior^7^ and the environmental conditions they encounter en route^8^,^9^,^10^,^11^. The availability of such datasets provides new opportunities for associating movement patterns, their causing factors and the resultant costs.

By climbing in rising air columns (thermals), soaring-gliding birds utilize energy from the environment; thereby dramatically decrease movement costs, compared with flapping flight^12^,^13^,^14^. Yet, despite a large and constantly increasing body of research on both interspecific^15^,^16^,^17^,^18^,^19^ and intraspecific^11^,^20^ differences in flight performance among soaring-gliding birds, only few studies have explored the role of age-related experience. Moreover, even those that do^3^,^21^ have drawn indirect inference on age-related experience effects based on relatively coarse movement data and without information on bird behavior, energy expenditure or environmental factors.

The art of thermal soaring, for birds and glider pilots alike^22^, requires development of several skills and efficient decision-making mainly due to the variation in the timing and location of appearance of individual thermals, as well as in their intensity and corresponding size^23^. We consider three basic components required for mastering this art: (1) *Thermal selection* – Since thermals vary in their intensity, duration, and shape across time and space, it is important for the bird to select an efficient path in terms of climbing in thermals, and decide which thermals to utilize. (2) *Thermal centering* – Once a bird can recognize and select favorable thermals, it must adjust its speed, banking angle (i.e., the angle at which the bird is inclined along its longitudinal axis with respect to the plane of its curved path), circling radius and maneuvering within a thermal in order to best utilize the strongest updrafts. Glider pilots consider this challenge “centering a thermal”, as updraft intensity exponentially declines when moving away from the core area; circling in a steeper banking angle decreases circling radius around the core but increases sink rate (relative to airflow) experienced by the individual. (3) *Inter-thermal gliding and airspeed selection* – This last component of mastering soaring-gliding flight is the bird’s ability to choose an optimal gliding airspeed between thermals in order to maximize cross-country speed^24^. More specifically, birds are expected to glide in a risk-sensitive manner according to the interplay between morphology and thermal conditions. The Risk-Averse Flight Index (RAFI) which is the ratio of actual to theoretical risk-averse gliding airspeed in inter-thermal gliding measures the level of risk aversion, hence more risk-prone flight with faster sink are indicted by lower RAFI values^17^. These components may have consequences on larger scale properties of movement and behavior, such as the efficiency of soaring-gliding flight and the tendency of individuals to use flapping flight. Soaring-gliding efficiency is defined as the inter-thermal displacement gained per given climbing time while soaring in thermals^25^,^26^, whereas, flapping flight has a dramatic effect on energy balance because it requires high energy expenditure compared to soaring-gliding flight^27^.

We studied foraging movements of Eurasian griffon vultures (*Gyps fulvus*), which rely heavily on thermal soaring (and also linear soaring at orographic uplifts)^28^ to minimize energy expenditure^29^ and typically forage in groups. To elucidate how experience affects thermal soaring performance, we measured age-related differences in soaring-gliding flight performance at high spatial and temporal resolution. Thermal soaring requires advanced skills and efficient decision making in relation to the above-mentioned basic challenges of soaring flight. We hypothesized that inexperienced juvenile vultures in their first two months after fledging would exhibit inferior thermal soaring performance compared with adult vultures having flight experience of at least five years. More specifically, we predicted that adults and juveniles, who typically forage in mix-aged groups, will exhibit similar thermal selection capacity. Adults, however, will exhibit higher climb rates in thermals compared to juveniles because thermal centering, in particular, is a difficult task to accomplish. Furthermore, to investigate the potential consequences of age-related differences in thermal soaring performance, we also examined age-related differences in larger-scale properties of the observed foraging trips and predicted that adults will exhibit higher soaring-gliding efficiency, less flapping flight, and hence lower energy expenditure during flight.

## Materials and Methods

### The study species and site

The Eurasian griffon vulture (*Gyps fulvus*; Hablizl 1783) is a long-lived, highly mobile, obligatory scavenger with social foraging skills^28^,^30^,^31^. In Israel, the breeding season usually spans from January to July, incubation lasts ~55 days, nestling rearing is ~110 days from hatching, and the post-fledging dependence period may last until September^30^,^32^. The local population in the Negev area (31°N 35°E) relies mainly on food supplied in an array of 25 supplementary feeding stations over an area of roughly 4,000 km^2^ by the Israel Nature and Parks Authority.

### Capture and measurements

As part of long-term monitoring efforts carried out by the Israel Nature and Park Authority, free-ranging vultures were captured outside the breeding season using a standard walk-in trap. Individuals were fitted with a 90-g GPS transmitters (E-Obs GmbH; Munich, Germany) weighted 1.5 ± 0.1% of the bird’s body mass below the recommended 3% for avian telemetry^33^ with a silicon harness covered with a Teflon ribbon (Bally Ribbon Mills, Pennsylvania, USA) in a backpack configuration. No adverse effects on behavior, neither breeding nor survival rate, were observed during the study. Capturing efforts and transmitter deployments were approved by the Israel Nature and Parks Authority and were in accordance with the ethics guidelines of the Hebrew University of Israel (NS-07-11063-2). Each of the tagged individuals was photographed on a scaled background in order to estimate wing span, wing area, aspect ratio (i.e., ratio of the square of wing span to wing area) and weighed in order to obtain wing-loading (i.e., ratio of mass to wing area). Measurements were done using ImageJ software (http://rsb.info.nih.gov/ij/).

### Data collection

GPS data-loggers provided accurate three-dimensional positioning (longitude, latitude and altitude), and an embedded tri-axial accelerometer supplying acceleration (ACC) data at 10 Hz per axis over 3.8 second intervals. GPS\ACC sampling effort had a diurnal duty cycle and the unit was activated for 13 hours on each day (6:00 to 19:00 local time; GMT+2). Sampling intervals for GPS were 1 second when the measured ground speed was above the in-flight threshold (2 m/s), and 600 seconds when the measured ground speed was below the in-flight threshold, and 600 and 60 seconds for ACC at the same scenarios.

### Data analysis

Daily paths were described by standard measures, including travel distance (sum of distanced between samples across the day), maximum displacement and straightness index (maximum displacement divided by travel distance until maximum displacement)^34^. Vertical speed was calculated as the difference in the measured altitude above ground level between sequential samples smoothed over a 5-second time window by a robust version of weighted local regression that assigns lower weight to outliers. ACC data during flight were classified using a supervised learning algorithm to identify flapping and soaring flight modes based on a validated dataset of observations in the field^7^,^34^,^35^.

### Environmental data analysis

Track annotation with environmental data were achieved by running the Regional Atmospheric Modeling System (RAMS)^36^. The European Centre for Medium-Range Weather Forecasts reanalysis data (ECMWF; http://www.ecmwf.int/) were used for RAMS model initialization and for forcing of meteorological conditions at the domain boundaries. Input variables were sea surface temperature, radiation, land-use and topographic data. Output variables included U (west-east) and V (south-north) components of the wind vector, and turbulent kinetic energy (TKE, a proxy of thermal intensity). The model was applied using three nested grids with the finest horizontal grid mesh of 1 km^2^ and vertical resolution increasing from 50 m (near ground) to 1000 m (at elevations over 9.8 km). Model data were saved at a temporal resolution of 5 minutes and coupled with interpolation for each location of a tagged individual^10^,^11^,^17^. For each point of the track we used the U and V wind components, which were combined in a single wind vector incorporating the strength and the direction of the wind, from which wind support (the wind component in the direction of travel) and side-wind (the wind component perpendicular to the direction of travel)^35^ components were obtained. Airspeed (velocity relative to the surrounding air) was calculated by subtraction of the wind vector from the ground speed vector of the bird^9^,^37^,^38^.

### Track segmentation

**Figure 1:**
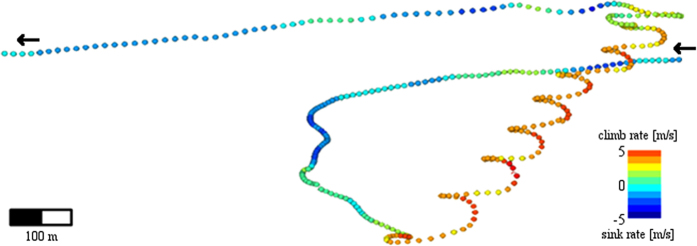
An example of a flight path of an adult vulture. Flight alternating between gliding (blue sections) and thermal soaring (ranging from yellow to red) modes. Flight took place on August 24^th^, 2013, in the Negev Desert. See Movie S1 for an animation of a daily flight.

The track was segmented to different flight modes (gliding, thermal soaring and linear soaring) in two stages. First, we identified thermals by searching for self-intersections (indicating loops or circles) of the path in two dimensions, excluding altitude. Such segments lasting more than 45 seconds and showing a positive altitude change were defined as a thermal soaring. Second, we identified gliding and linear soaring segments by locating segments with a similar vertical speed trend (positive or negative, respectively) with a chosen threshold of 90% of samples maintaining the same trend. In order to find the transition point between adjacent segments, the edges of each segment were trimmed as long as the proportion of samples with the expected trend increased. Following the track segmentation, we characterized the different movement modes (Fig. 1), using the time, duration, location, altitudinal change, travel distance and average speeds (vertical, horizontal and angular) of each segment. Wind support and side-wind were estimated for gliding segments only.

### Quantifying flight performance

The fine resolution of the data provided the opportunity to describe soaring behavior yet limited our ability to observe a gradual process of learning due to the tradeoff between the sampling interval and the overall duration of tracking. We therefore use two distinct age categories of juveniles in their first two months after fledging, and adults having flight experience of at least five years. *Thermal selection* was estimated by examining the mean TKE at one kilometer scale associated with each thermal. As the exact thermal locations and times are considered variable^23^, and we do not expect that thermals will develop at the same time and location as in the model, because the TKE gives a more regional indication relevant for the vultures’ decision making in a larger area. *Thermal centering* was estimated by examining the climb rate. To evaluate the relationship between wind shear and the difference in climb rate between adults and juveniles climb rate, we considered three alternative effects: No effect, a linear effect, and a hump-shaped effect, and chose the best fitting model using Akaike’s information criterion with a correction for small sample size (AICc)^39^. For each thermal soaring event we characterized flight versus wind direction, a circular measure ranging between headwind (0 degrees) and tailwind (±180 degrees), taking into account the leeside and windward side of the thermal by separately analyzing clockwise and counter-clockwise circling events in order to quantify the effect of the wind on the individual. *Inter-thermal gliding airspeed selection* was defined for each gliding segment, using the Risk-Aversion Flight Index (RAFI) to assess the tendency of birds to glide slowly but safely (near best glide speed - highest ratio of airspeed to sink speed; high RAFI values) or fast (by adjusting airspeed to the rate of ascent at the soaring phase) but with risk of grounding or switching to flapping flight; low RAFI values)^17^.

Soaring-gliding efficiency was used as a proxy for time minimization, and was calculated as the distance travelled when gliding divided by the preceding thermal soaring duration. Daily flapping proportion was estimated as the proportion of samples within the day that were classified as flapping. To estimate energy expenditure, we calculated the Overall Dynamic Body Acceleration (ODBA)^40^. ODBA was previously linked with energy expenditure in griffon vultures, heart rate and ODBA were, 2–3 and 4–5 times higher during flapping compared to gliding flight, respectively^29^. Moreover, heart rate in the same species was shown to be correlated with oxygen consumption in lab conditions^13^. We note that we do not use ODBA to estimate absolute energy expenditure but for comparative purposes, assuming that age-related differences in the match between ODBA and energy expenditure are relatively minor.

In order to focus on foraging flights, long-range movements were excluded based on the distance from the mode main roost of the population, the daily travel distance (>200 km) and the straightness of the daily path (>0.7)^25^,^41^. Over the foraging track we estimated the distance travelled when gliding divided by the preceding thermal soaring duration (assuming higher values represent better time minimization), the proportion of flapping flight measurements and the mean ODBA during the daily flight. ANCOVA was used to determine the effect of wind shear on circling radii. Due to the small sample size we used Mann-Whitney-Wilcoxon (MWW) tests. Data were analyzed using Matlab2013a (MathWorks Inc, Natick, MA, USA).

## Results

During a period of 4 months (July 2013 – October 2013) we collected data on the movements of 8 juveniles (0–2 months from fledging) and 9 adults (older than 5 years). The tracks of these birds lasted 12 ± 2 days (mean ± SE) totaling ~3 million GPS points in flight, maintaining a constant sampling effort across the different age classes (MWW; N = 9 adults, 8 juveniles; *U* = 62, *P* = 0.36). The track segmentation procedure yielded hundreds of gliding and thermal soaring events per individual and only tens of linear soaring events (Table 1), suggesting predominant use of convective thermals.

### Thermal selection and centering

**Figure 2:**
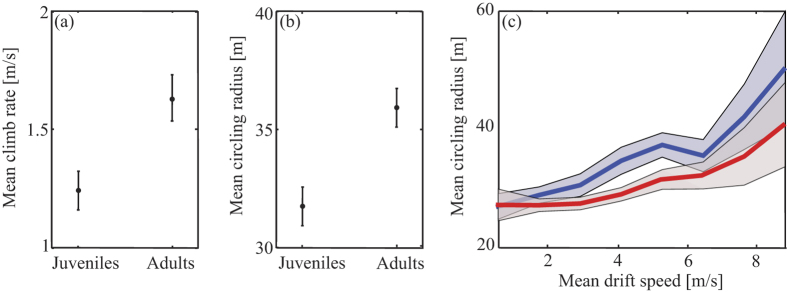
Age-related differences in thermal centering. (**a**) Climb rates in thermals of juvenile and adult vultures differed, with juveniles having lower climb rates (*U* = 85, *P* = 0.003). (**b**) The circling radius of juveniles was smaller (*U* = 79, *P* = 0.02). (**c**) No age-related differences in circling radii under low wind shear conditions were observed, but greater circling radii were found for adults (blue) compared with juveniles (red) under intermediate wind shear conditions. Grayish areas indicate SE. Values in panels a and b are mean ± SE.

**Figure 3:**
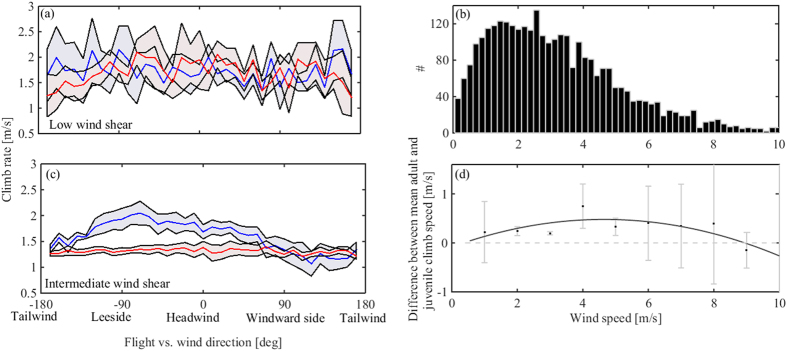
Thermal centering in different wind conditions. (**a**) Climb rates under low wind shear conditions (<2 m/s) and under strong wind shear conditions (>6 m/s) were not significantly different between age classes, but (**c**) under intermediate wind shear conditions (2–6 m/s) adults (blue) exhibited higher climb rates than juveniles (red). (**b**) Histogram of the estimated horizontal wind speed in thermal soaring events by RAMS. (**d**) The difference between mean climb rate of juveniles and adults as a function of lateral wind speed. Best fitting model among a hump-shaped model (second-order polynomial, solid black) and no difference among age classes (y = 0, dashed grey) are presented. Error bars represent mean ± SE.

We found no age-related differences in the TKE which served a estimator for *thermal selection* (adults: 1.2 ± 0.34, juveniles: 0.85 ± 0.2; *U* = 37, *P* = 0.2; mean ± SE). During the first months of their life, vultures showed lower mean climb rates in thermals (adults: 1.6 ± 0.17, juveniles: 1.26 ± 0.06; *U* = 85, *P* = 0.003; Fig. 2a) and smaller circling radii compared with adult birds (adults: 35.9 ± 0.8, juveniles: 31.8 ± 0.7; *U* = 79, *P* = 0.02) (Fig. 2b). The observed differences were not related to age-related variation in wing-loading (adults: 9 ± 0.16, juveniles: 8.7 ± 0.31; *U* = 21, *P* = 0.42) or aspect ratio (adults: 7.61 ± 0.06, juveniles: 7.54 ± 0.05; *U* = 12, *P* = 0.46, respectively). As expected, wind shear conditions measured by the RAMS were diverse (Fig. 3b). In both low (<2 m/s) and strong (>6 m/s) wind shear conditions no age-related differences were found in circling radii, but at intermediate wind shear conditions (2–6 m/s) adults exhibited larger circling radii (ANCOVA; *F* = 4.89, *P* = 0.03; Fig. 2c). In low wind shear conditions (<2 m/s; Fig. 3a), climb rate showed no variation, but in intermediate wind shear conditions (2–6 m/s), adults had higher climb rates at the leeside of the wind and similar values at the windward side (Fig. 3c). A hump-shaped response was the best fitting for the calculated difference in climb rate between adults and juveniles over different wind shear values (Table 2, Fig. 3d).

### Inter-thermal gliding airspeed selection

We found no age-related differences in the Risk-Aversion Flight Index (RAFI; adults: 0.76 ± 0.05, juveniles: 0.69 ± 0.05; *U* = 36, *P* = 0.42). During gliding segments, juveniles experienced marginally significant lower wind support (adults: 0.45 ± 0.35, juveniles: 0.03 ± 0.04; *U* = 41, *P* = 0.06) and similar side-wind (adults: 0.32 ± 0.17, juveniles: −0.01 ± 0.03; *U* = 39, *P* = 0.76).

### Soaring-gliding efficiency and energy expenditure

**Figure 4:**
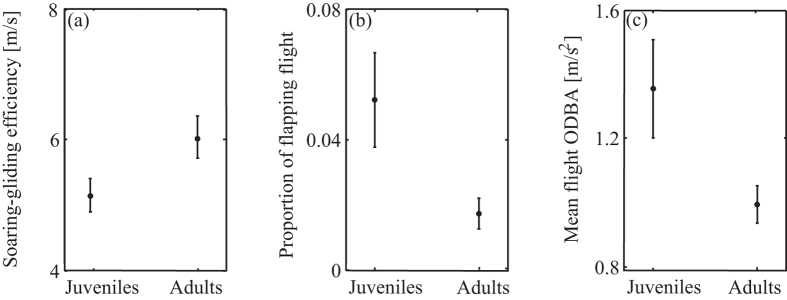
Soaring-gliding efficiency and energy costs. (**a**) Juveniles exhibited lower soaring-gliding efficiency (*U* = 70, *P* = 0.006), (**b**) used flapping more frequently (*U* = 14, *P* = 0.025), and (**c**) had higher ODBA values during foraging flights (*U* = 13, *P* = 0.016). Values are mean ± SE.

Foraging trips of adult and juvenile birds showed similar general characteristics, with no observed differences in daily travel distance (adults: 155 ± 14, juveniles: 142 ± 10; *U* = 31, *P* = 0.46), daily path straightness (adults: 0.2 ± 0.02, juveniles: 0.23 ± 0.02; *U* = 47, *P* = 0.48) and daily maximum displacement (adults: 28.5 ± 2.3, juveniles: 31.6 ± 3; U = 24, P = 0.7). However, juveniles showed lower soaring-gliding efficiency (adults: 6 ± 0.4, juveniles: 5.2 ± 0.3; *U* = 70, *P* = 0.006; Fig. 4a) and expended more energy per time unit, as inferred from their higher Overall Dynamic Body Acceleration (ODBA) during flight (adults: 0.99 ± 0.06, juveniles: 1.34 ± 0.15; *U* = 17, *P* = 0.01; Fig. 4c). This result can be attributed, at least partly, to the higher proportion of flapping flight in juveniles compared with adults (adults: 0.02 ± 0.003, juveniles: 0.05 ± 0.01; *U* = 13, *P* = 0.016; Fig. 4b).

## Discussion

Vultures forage in mixed-age groups in search of unpredictable and sparsely distributed food resources^42^,^43^,^44^. This foraging strategy is likely to mask differences in flight performance among individuals, explaining our findings that the ability of inexperienced juvenile vultures to select favorable thermals and to climb thermals in weak lateral winds in their first months of life is comparable to that of adult birds experienced with thousands of foraging days. However the difference observed at intermediate wind shear could be explained, at least in part, by problems encountered by juvenile birds in centering thermals under harsh circumstances, such as drifted thermals due to intermediately strong lateral winds. Presumably, adult birds, in contrast, exhibit a rather systematic centering method (Fig. 1). They progress “patiently” with a tailwind until entering a zone of strong updrafts, make a sharp turn with substantial elevation gain at the lee side of the thermal, then level again at tailwind bearing. Examination of large-scale properties of flight patterns revealed that juveniles had a lower soaring-gliding efficiency, higher proportion of flapping flight, and higher energy expenditure during flight, as inferred from the higher ODBA, compared with adults.

Our finding that adult vultures climb faster than juveniles despite similar TKE and wing-loading suggests that their faster climb rates reflect a better centering capacity within the thermal rather than selection of stronger thermals. Thermal soaring birds can climb faster by centering close to the thermal core, at the position of maximal updraft, but this requires a smaller circling radius achieved by a steeper banking angle. Yet, circling at a steep banking angle entails a cost of lower lift produced by wings, hence less efficient upward movement in relation to the rising air mass^45^. The finding that adults have a larger circling radius than juveniles might suggest that adults make more optimal choices considering this trade-off: they prefer to fly at a less steep banking angle, hence enjoying a higher lift, despite the weaker updrafts farther from the core of the thermal. We suggest an alternative explanation, which corresponds well with the finding that the circling radius of adults tends to increase with stronger thermal drift (Fig. 2): the larger circling radius of adults is a by-product of their systematic centering method described above, maintaining lift during the lengthier leveling phase and attaining a rapid elevation gain by turning sharply upon reaching the core of the thermal. This method provides better climb rates for adults compared with juveniles at increasing lateral wind speed. The observed hump-shaped pattern implies that the ability of vultures, both juveniles and adults, to exploit drifted thermals decreases in more extreme wind conditions, up to a certain threshold (Fig. 3).

The lower flight performance of juvenile vultures corresponds to lower performance of juvenile seabirds revealed at larger spatiotemporal scales. During the first months after fledging, wandering albatrosses showed inferior foraging performance compared to sub-adults and adults^46^. Juvenile brown boobies improved their flight abilities during the first month after fledging^47^, and juvenile European shags compensated for poor foraging success by investing a higher proportion of the day in foraging in the first months after independence, compared with adults^48^. S. Rotics *et al.*^5^ link lower daily displacement of juvenile white storks with extensive use of flapping flight, resulting in low survival rates of juveniles. Here we further develop the link between the patterns observed at coarser scales of low performance of juveniles to the proposed specific mechanism underlying the observed variation in soaring-gliding birds. While in this work we compare flight performance patterns of juveniles and adults, future research collecting continuous movement data on free-ranging individuals along the critical period of flight ontogeny may provide valuable insights and link individual background, accumulated experience, the observed movement path and survival.

A central consideration in inferring the mechanisms underlying movement patterns is the critical role of internal motivation, which could change dramatically in the life of individuals, affecting the individual’s decisions and the resultant movement path^6^. To minimize variation in the (unknown) motivation of individual juveniles versus adults, we focused here on foraging movements, and analyzed movement tracks executed within a short period during which adults and juveniles move across comparable spatial scales and the weather conditions are pretty stable. At longer time periods, juveniles gain experience and are likely to differ in their motivation from adults who tend to be philopatric while juveniles tend to cover greater distances to explore the environment as part of natal dispersal^49^, excursions, or migration^50^. Such long movements may magnify the cost of inefficient thermal soaring, giving rise to a strong selection pressure for rapidly learning efficient soaring. Difficulties in food handling^47^ and inferior navigation capacity in an unfamiliar environment^21^ could augment these selection pressures, accounting for the general tendency of high mortality rates early in life^51^.

High-resolution movement datasets obtained from free-ranging wild animals, coupled with data on their behavior and the environment, were instrumental in addressing basic questions that have been beyond reach until now, such as the role of experience in basic functional tasks. We found that adult vultures performed better than juveniles under challenging environmental conditions of relatively strong wind when thermal centering is difficult. We also found considerably lower flight efficiency of inexperienced juveniles at daily time scales, both in terms of energy expenditure and time allocation. With such technologies now in reach, we are getting closer to the actual spatial and temporal scales at which decision making occurs. This approach could be extended to address other key questions (e.g., behavioral response to unusual environmental conditions) at the interface between ecology, biomechanics, environmental modeling and behavior.

## Additional Information

**How to cite this article**: Harel, R. *et al.* Adult vultures outperform juveniles in challenging thermal soaring conditions. *Sci. Rep.* **6**, 27865; doi: 10.1038/srep27865 (2016).

## Supplementary Material

Supplementary Information

Supplementary Movie S1

## Figures and Tables

**Table 1:** Summary statistics of soaring-gliding movement modes (gliding, thermal and linear soaring) after a segmentaion procedure.

Movement mode	Events (#)	Duration (s)	Vertical speed (m/s)	Altitudinal change (m)
Gliding	422 ± 77	192 ± 6	−0.75 ± 0.03	−210 ± 10
Thermal soaring	273 ± 45	150 ± 5	1.4 ± 0.06	266 ± 12
Linear soaring	21 ± 5	80 ± 2	1.8 ± 0.07	218 ± 15

The number of recorded events and mean values of duration, vertical speed and altitudinal change for each individual are presented (mean ± STD).

**Table 2:** The effect of wind shear conditions on age-related differences in climb rates in thermal soaring.

Polynomial fit	Function	Adjusted R^2^	AICc	P value
No effect	*y* = 0.225	0.00	−0.22	
Linear effect	*y* = 0.041*x* + 0.019	0.16	0.44	0.58
Hump-shaped effect	*y* = −0.025*x*^2^ + 0.29*x* − 0.445	0.70	−5.44	0.05

Three alternative models were considered: No effect, a linear effect and a hump-shaped effect. The best fitted model for each scenario is described using AICc and adjusted R^2^ estimation of goodness of fit.
