# Supplementary material for: Adult vultures outperform juveniles in challenging thermal soaring conditions
Source: Sci Rep. 2016 Jun 13;6:27865. doi: 10.1038/srep27865 (PMC4904409; doi:10.1038/srep27865)
Supplement: Supplementary Information [file srep27865-s1.docx]

**Supplementary material**

**Adult vultures outperform juveniles in challenging thermal soaring conditions**

**Roi Harel, Nir Horvitz, Ran Nathan**

Movie S1. The animation shows soaring-gliding flight of Eurasian griffon vultures in the Negev Desert, Israel. The color of the circles represents vertical flight speed alternating between gliding (blue sections) and thermal soaring (ranging from yellow to red), and the radius represents instantaneous ground speed. During the first 15 seconds one individual is visible and then two additional vultures join the flight. The animation runs at 45× time lapse.
